# Polymeric-Based Theranostic Nanocarriers of Neuroprotective Drugs: Development, Imaging, and Bioanalysis

**DOI:** 10.1021/acs.molpharmaceut.5c00170

**Published:** 2025-06-15

**Authors:** Krzysztof Szczepanowicz, Magdalena Procner, Marta Szczęch, Natalia Łopuszyńska, Danuta Jantas, Magdalena Regulska, Monika Leśkiewicz, Krzysztof Jasiński, Kamil Stachurski, Lilianna Szyk-Warszyńska, Adam Roman, Władysław Lasoń, Władysław P. Węglarz, Piotr Warszyński

**Affiliations:** † Jerzy Haber Institute of Catalysis and Surface Chemistry, Polish Academy of Sciences, Niezapominajek 8, Kraków 30-239, Poland; ‡ 69714 Maj Institute of Pharmacology, Polish Academy of Sciences, Smętna 12, Kraków 31-343, Poland; § Henryk Niewodniczański Institute of Nuclear Physics, Polish Academy of Sciences, Radzikowskiego 152, Kraków 31-342, Poland

**Keywords:** nanocarriers, neuroprotection, theranostic, blood−brain barrier, magnetic resonance imaging, optical imaging

## Abstract

Inefficient delivery of neuroprotective drugs to their target sites remains a major impediment in the treatment of neurodegenerative disorders. Therefore, our research was focused on a new strategy for the preparation of polymeric-based theranostic nanocarriers of neuroprotective drugs. Polymeric theranostic nanocarriers of calcineurin inhibitors, Cyclosporin A (CsA) and Tacrolimus (FK506), as potential neuroprotective agents, were prepared via the self-emulsification solvent evaporation (SESE) method with the combination of a layer-by-layer technique. For magnetic resonance imaging, gadolinium-labeled poly-l-lysine (PLL-Gd) was used, while for optical imaging, rhodamine-labeled poly-l-lysine (PLL-ROD) was used. Developed nanocarriers were characterized for their properties: the size was below 250 nm, the encapsulation efficiency was ∼100%, and they could serve as transport devices for therapeutic cargo and imaging compounds, e.g., distribution assessment. Developed nanocarriers were safe for tested cells (human neuroblastoma cells, primary neuronal cell cultures, and brain microvascular endothelial cells). Equally important, they willingly traversed the artificial blood–brain barrier. Our study demonstrated that the newly designed polymeric-based theranostic nanocarriers possess favorable physicochemical and biological properties and may serve as a useful platform for neuroprotective compound delivery.

## Graphical abstract

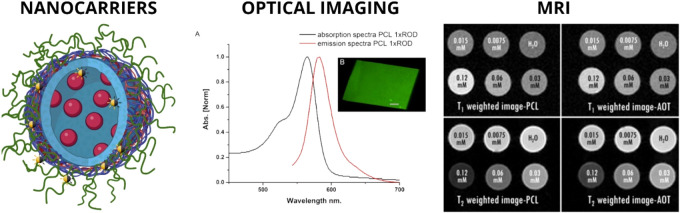

## 1 Introduction

The global incidence of central nervous system (CNS) disorders is increasing, yet CNS drug development faces considerable hurdles: high costs, prolonged clinical timelines, and frequent failures.^1^ A primary challenge for researchers treating CNS diseases is the ineffective delivery of neuroprotective substances to target brain areas. This critical limitation is largely imposed by the blood–brain barrier (BBB), a complex system of transport proteins and highly regulated cells. It can be considered an active obstacle separating the circulation from the brain tissue. The barrier’s main task is to protect neurons from toxins or pathogens and to remove waste products from the brain; therefore, it plays a crucial role in maintaining homeostasis. Only small (<1 kDa), lipophilic molecules can pass the BBB; hence, permeation of most drugs, imaging contrast agents, proteins, and nucleic acids is prevented.^2^ From the clinical point of view, this relative impermeability is critical and limits the ability of therapeutic agents to achieve appropriate concentrations in the brain tissue. The described phenomenon is based on two predominant reasons—the formation of a tight junction (TJ) restricting the paracellular transport and the active transporting out of the BBB to the bloodstream by efflux pumps.^3,4^ Since the BBB is very difficult to cross, the development of specialized drug delivery systems for the effective treatment of CNS disorders, which allow for transportation through the BBB, is currently being extensively investigated. One of the approaches to overcome these drawbacks relies on nanotechnologies, which emerge with innovative tools (including drug nanocarrier systems) for therapeutic and diagnostic purposes. Nanocarriers (NCs) can simultaneously encapsulate both therapeutic compounds and imaging agents. Their surface can be functionalized with targeting ligands and cloaking agents, such as polyethylene glycol (PEG), to achieve prolonged circulation.^5–7^ Consequently, such nanotheranostics represent a cutting-edge, personalized therapeutic approach. They are capable of enabling accurate diagnosis alongside the effective and targeted delivery of therapeutics across the BBB to injured regions of the CNS. As delivery systems, theranostic NCs can prevent drug degradation and nonspecific binding, thereby reducing potential toxicity while also facilitating drug passage across the BBB through their specific pharmacological actions. Furthermore, they can serve as diagnostic tools if traceable by imaging techniques, such as positron emission tomography (PET), computed tomography (CT), ultrasound, optical imaging (OI), and magnetic resonance imaging (MRI). A wide range of pharmaceutical NCs have been developed, including liposomes, solid lipid nanoparticles, micelles, dendrimers, and various other nanoparticles.^1,2,8–11^ Among them, polymeric nanoparticles represent promising tools for delivering neuroprotective actives as well as diagnostic agents, mainly due to their versatility, which comes from the ability to control physicochemical properties like size, size distribution (PDI), shape, and charge, and due to their biocompatibility, biodegradability, low cytotoxicity, and ability to cross biological barriers.^2,12–15^ Moreover, polymeric nanoparticles can be easily functionalized, e.g., by targeting ligands (which may help to increase the local concentration of the therapeutic at the site of action).^5^ A powerful method for functionalizing polymeric nanoparticles is the layer-by-layer (LbL) technique, which relies on the sequential adsorption of charged molecules.^16–22^ The LbL method offers significant advantages, notably, its ease of manipulation and its inherent multifunctionality. This multifunctionality stems from the capacity to create a multilayer shell incorporating diverse functional charged species, including antibodies, aptamers, or inorganic nanoparticles.^23–33^

Based on the challenges mentioned above, the present study aimed to develop a new strategy for the preparation of polymeric-based theranostic NCs of neuroprotective drugs. Polymeric NCs of selected drugs were prepared via the self-emulsification solvent evaporation (SESE) method combined with the layer-by-layer technique. Calcineurin inhibitors, Cyclosporin A (CsA) and Tacrolimus (FK506), as potential neuroprotective agents were selected for encapsulation in NCs. Afterward, NCs were functionalized for OI and MRI. OI, or fluorescence imaging, is highly regarded in biomedical applications due to its ease of use and cost-effectiveness compared to other techniques.^34^ A substantial number of fluorescence probes have been engineered, offering high sensitivity, real-time detection capabilities, and operational simplicity in biological imaging. Notably, rhodamine dyes have found extensive application in biotechnology as fluorescent markers and for biomolecular detection, which is attributed to their favorable optical and physical attributes. Therefore, rhodamine was selected for the labeling of polymeric NCs intended for OI. For noninvasive imaging, MRI is another powerful modality. Gadolinium-based contrast agents constitute the overwhelming majority of those currently employed.^35,36^ Thus, Gd-DTPA was selected to label polymeric NCs for MRI.

Due to the fact that the BBB is an extremely complex and challenging system and hence their permeability is crucial for drug delivery development, the permeation studies seem to be exceedingly important. Depending on which particular functional aspect is necessary to investigate, one of several *in vitro* models of the BBB can be used such as endothelial and epithelial cell lines (e.g., human umbilical endothelial cells (HUVECs), Madin–Darby canine kidney (MDCK)) or primary cultures of brain endothelial cells.^37^ Among others, the immortalized human brain microvascular endothelial cell line (hCMEC/D3) seems to be suitable for studying the barrier permeability for neuroprotectants and drug carriers. This well-characterized *in vitro* model of the BBB closely imitates the *in vivo* BBB phenotype and is also replicable, easy to grow, and can express the TJ and efflux transporters.^38^ Therefore, in this study, we evaluated the capability of designed NCs to pass through the hCMEC/D3 cell monolayer to test if they can be considered promising platforms for drug delivery. The general scheme of the study is presented in Figure 1.

**Figure 1:**
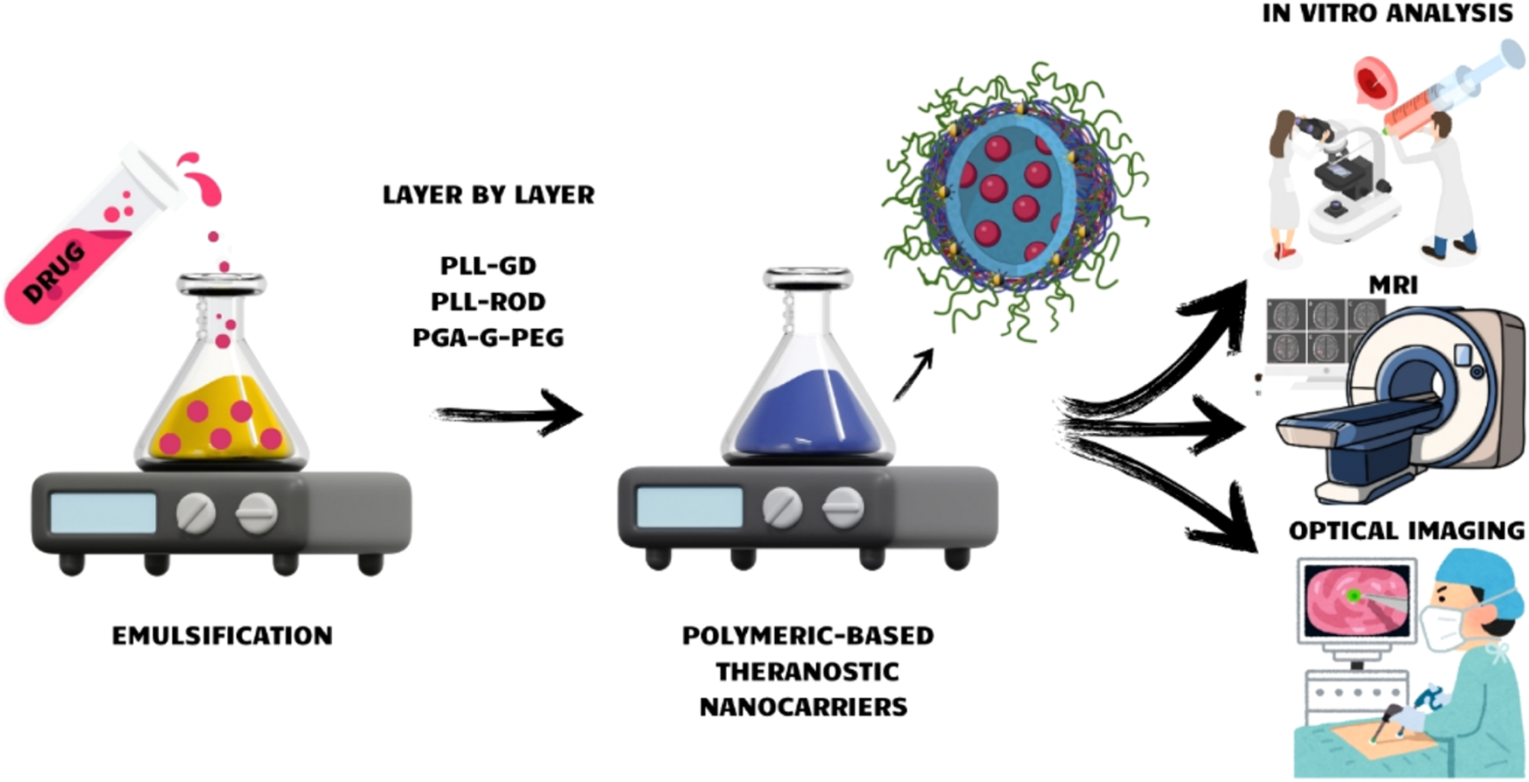
General scheme of the presented work (created in https://BioRender.com and Canva.com).

## 2 Materials and Methods

### 2.1 Materials

#### 2.1.1 Chemicals

Polycaprolactone (PCL, average *M*_w_ ca. 14 kDa), poly-l-lysine hydrobromide (PLL, average *M*_w_ 15–30 kDa), poly-l-glutamic acid sodium salt (PGA, average *M*_w_ 15–50 kDa), sodium chloride (NaCl), and lissamine rhodamine B sulfonyl chloride (ROD) were purchased from Sigma-Aldrich Chemie GmbH (Germany). Docusate sodium salt (AOT) was a commercial product of Cytec Solvay (Poland). Gadolinium-labeled poly-l-lysine (PLL-Gd, *M*_w_ 22 kDa) was acquired from BioPAL, Inc. The pegylated polyanion, PGA-*g*-PEG (g ∼30% and PEG, *M*_w_ ∼5 kDa), was synthesized according to the protocol described previously,^39^ while the rhodamine-labeled polycation (PLL-ROD) was synthesized via coupling of the fluorescent marker, according to the protocol described in^40^. Cyclosporin A (CsA) and Tacrolimus (FK506) were received from Selleck Chemicals. Chloroform and ethyl alcohol (anhydrous, 99.8%) were purchased from Avantor Performance Materials Poland S.A. Ultrapurified water was generated using a Direct-Q5 UV purification system (Millipore, Poland). All chemicals and solvents were of analytical grade and employed without further purification.

#### 2.1.2 Materials for Cell Culture and Cytotoxicity Tests

The human neuroblastoma SH-SY5Y cell line was purchased from the American Type Culture Collection (CRL-2266, ATCC, Manassas, VA). The immortalized hCMEC/D3 cell line (human cerebral microvascular endothelial cells/clone D3) was provided by Sigma-Aldrich Chemie GmbH (Germany). High-glucose Dulbecco’s modified Eagle’s medium (DMEM), FluoroBrite DMEM, DPBS, heat-inactivated fetal bovine serum (FBS), penicillin/streptomycin mixture (10,000 U/mL), trypsin/EDTA solution, N-2 supplement, chemically defined lipid concentrate, HEPES, and human basic fibroblast growth factor (bFGF), Neurobasal A (w/o phenol red), and supplement B27 (w/o antioxidants) were obtained from Gibco (Life Technologies Ltd., Paisley, UK). The cytotoxicity detection kit (LDH) and cell proliferation reagent WST-1 were provided by Roche Diagnostics GmbH (Mannheim, Germany). Ascorbic acid, Triton X-100 solution, dimethyl sulfoxide (DMSO) ReagentPlus ≥99.5%, and hydrogen peroxide solution 30% (w/w) in H_2_O were obtained from Sigma-Aldrich Chemie GmbH (Taufkirchen, Germany). Endothelial cell basal medium (EBM-2) and Cultrex rat collagen I (lower viscosity) were obtained from Lonza Group Ltd. (Basel, Switzerland) and R&D Systems (Minneapolis, MN), respectively. The 24-well plates with collagen-coated transwell inserts (pore size 1 μm, polyethylene terephthalate) were purchased from Corning Life Sciences (New York). All reagents were applied without further purification. All concentrations given in the text are the final values received after diluting the reactants.

### 2.2 Methods

#### 2.2.1 Preparation of Polymeric-Based Theranostic NCs of Selected Neuroprotective Drugs

Polymeric-based theranostic NCs of selected neuroprotective drugs were prepared according to the reported protocols.^36^ Briefly, polymeric (solid) and nanoemulsion (liquid) cores of the NCs were prepared by using a nanoemulsion template formed by self-emulsification combined with the solvent evaporation (SESE) method. The nanoemulsion oil phase consisted of an anionic surfactant (AOT) dissolved in chloroform that provided the liquid core. In contrast, the solid core was made of a polymer (PCL), which was codissolved with AOT in the oil phase. The concentration of PCL in the oil phase was 10 g/L, while that of AOT was 340 g/L in both cases of the nanocores’ formation. The nanoemulsion aqueous phase consisted of a polyelectrolyte (PLL) solution in 15 mM NaCl. The optimal PLL concentration was 0.2 g/L at natural pH, i.e., without adjusting the pH.^22,36^ The nanoemulsions were formed by adding 0.1 mL of the oil phase to 200 mL of the aqueous phase. In the case of polymeric core formation, the oil phase was first mixed with 10 mL of anhydrous ethanol and then added to the aqueous phase. The emulsification process was carried out with continuous mixing by using a magnetic stirrer at 300–500 rpm. Continuous stirring after evaporation of the organic solvent led to the formation of polymeric or nanoemulsion cores. Throughout this process, the final chloroform concentration was carefully controlled and did not exceed 0.04 mg/L.^39^ To prepare the neuroprotectant-loaded polymeric-based theranostic NCs, the selected neuroprotective substances (CsA or FK506) were encapsulated into both types of nanocores (solid and liquid). Specifically, each drug was dissolved in the proper oil phase before emulsification. Their concentration was optimized by changing the amount dissolved in the oil phase. Concentrations of the components of nanoemulsions are summarized in Table 1.

**Table 1:** Concentrations of the Nanoemulsion Components

	oil phase (chloroform) 0.1 mL	conc. (g/L)	aqueous phase (15 mM NaCl) 200 mL	conc. (g/L)
liquid core	AOT	340	PLL	0.2
	FK506	50		
	cyclosporine A	50		
solid core	AOT	340	PLL	0.2
	PCL	10		
	FK506	50		
	cyclosporine A	50		

#### 2.2.2 Evaluation of the Drug Encapsulation Efficiency

The drug entrapment efficacy (DEE) in the polymeric and nanoemulsion cores was determined by centrifugation. Namely, the final suspension of drug-loaded nanocores was centrifuged using Amicon Ultra-4 centrifugal filters, Ultracel-3K (Merck Millipore Ltd., Cork, Ireland). The spin rate was 10,000 rpm at 25 °C, and the total centrifugation time was 60 min. The presence of free unencapsulated drugs in the filtrate was detected by UV–vis spectroscopy.

The percentage value of the DEE was calculated by the following formula:

1 $$\mathrm{DEE}=\frac{\left|\mathrm{drug}\right|\mathrm{total}-|\mathrm{drug}|\mathrm{supernatant}}{|\mathrm{drug}|\mathrm{total}}\times 100\%$$

where |drug|total is the total weight of the drug and |drug|supernatant is the weight of the free (unencapsulated) drug.

#### 2.2.3 Polymeric-Based NC Functionalization for Magnetic Resonance Imaging (MRI) and Optical Imaging (OI)

To prepare polymeric NCs for imaging modalities, multifunctional polyelectrolyte shells containing selected molecules were prepared. For MR imaging, gadolinium-labeled poly-l-lysine (PLL-Gd) was used, while for optical imaging, rhodamine-labeled poly-l-lysine (PLL-ROD) was used. The functionalized shell was formed on both nanocores (liquid, L and solid, S) using the LbL assembly approach and saturation technique. Specifically, the multilayer shells comprised the following polyelectrolytes: PGA (−) and PLL-Gd (+) for MRI, PGA and PLL-ROD (+) for OI, and PGA and PLL (+) as the corresponding references. In order to check the optimal structure for MRI measurements, shells with one and two layers of PLL-Gd were prepared. All types of multilayer shells were ended with PGA-g-PEG. The procedure of formation of the multilayer film by the saturation method was described in detail in our previous publications.^41–45^ Briefly, a fixed volume of the nanocore or NC suspension was added to various volumes of the oppositely charged polyelectrolyte solution under continuous stirring. Layer formation was monitored by zeta potential measurements, and the optimal amount of the polyelectrolyte was determined using the saturation technique.^22,43,45^ This iterative deposition of polyelectrolyte layers continued until the desired multilayer shell was achieved.

#### 2.2.4 Size, PDI, and Zeta Potential

The size (hydrodynamic diameter), polydispersity index (PDI), and zeta potential of the polymeric-based theranostic nanocarriers and their components were measured using a Zetasizer Nano ZS (Malvern Panalytical Instruments, United Kingdom), which employs dynamic light scattering (DLS) and electrophoretic light scattering (ELS) techniques. Measurements were performed in optically homogeneous polystyrene cells and folded capillary zeta cells, respectively. All measurements were performed at 25 °C, and each value was received as an average of at least three subsequent measurements with 20 runs.

#### 2.2.5 UV–Vis Spectroscopy

The drug encapsulation efficiency was evaluated by using UV–vis spectroscopy (UV-1800 spectrophotometer, Shimadzu Corporation, Kyoto, Japan).

#### 2.2.6 Cryo-SEM

The morphology of polymeric-based theranostic nanocarriers was analyzed using a cryo-scanning electron microscope (cryo-SEM), specifically a Jeol JSM-7600F field emission scanning electron microscope (FESEM, Jeol Ltd., Tokyo, Japan), following a previously described protocol^46^ or sample preparation; a droplet of the nanocarrier suspension was placed on a cold sample holder. This holder was then attached to a transfer rod and immediately frozen in liquid nitrogen using a Quorum PPT2000 cryopreparation stage (Polaron, Quorum Technologies, United Kingdom). The frozen sample was cryo-transferred under liquid nitrogen vapor to the cryo-unit chamber, where it underwent sublimation at −70 °C for 15–30 min until all visible ice crystals had disappeared. The sample was subsequently sputter-coated with a 5 nm layer of platinum. The specimen was then transferred to the cooled stage of the Jeol JSM-7600F field emission scanning electron microscope (FESEM) for subsequent imaging.

#### 2.2.7 Optical Imaging: Fluorescence Spectroscopy and Microscopy

Fluorescence emission spectra were measured to confirm the formation of rhodamine-labeled nanocarriers. Spectra were obtained using a Fluorolog-3 spectrofluorometer from Jobin Yvon Inc. with an excitation wavelength of 520 nm. The visualization of rhodamine-labeled NCs incorporated into agarose gel phantom was performed by Carl Zeiss LSM780 (Carl Zeiss, Jena, Germany) confocal microscopy (exc. 488 nm, filters 527–695, pinhole 58 μm, obj. 63 × 1.4 oil DIC M27).

#### 2.2.8 MR Imaging and Relaxometry

MR imaging and relaxometry of a series of dilutions of AOT- and PCL-based nanocapsules, with one and two layers containing incorporated PLL-Gd, were performed using the 9.4T BioSpec 94/20 preclinical MRI scanner (Bruker BioSpin, Germany), equipped with BGA60-S gradient coils and a 35 mm birdcage RF coil, controlled by Paravision 6.1 software. For the acquisition of the series of axial images of each sample with varied parameters, the RARE with variable repetition time TR (RARE VTR) and multislice, multiecho (MSME) imaging sequences were used. The RARE VTR sequence was used to determine the values of *T*_1_ relaxation times for individual samples. The imaging geometry parameters were as follows: layer thickness, 1 mm; matrix, 128 × 128; field of view, 3.0 × 3.0 cm. The echo time (TE) value was set to 7.0 ms. The number of *T*_1_ experiments for samples with a single layer of PLL-Gd was 10 and the effective repetition time TR values were in the 19.5–9989.5 ms range. For the corresponding reference samples, 12 repetitions were used with the effective TR in the 19.5–14989.5 ms range. For samples with double layers of PLL-Gd and the corresponding reference samples, the number of *T*_1_ experiments was 12 and the effective repetition time TR values were in the 19.5–14989.5 ms range. The MSME sequence with the following parameters was used to measure the *T*_2_ relaxation time: layer thickness: 1 mm; MTX: 128 × 128; FOV: 3.0 × 3.0 cm; TR: 10,000 ms. For samples with a single PLL-Gd layer, 35 echoes were used with TE values in the 30–1050 ms range, while for the corresponding reference series, 80 echoes were selected with TE values in the 50–4000 ms range. Similarly, for samples with double layers of PLL-Gd and the corresponding reference samples, the number of echoes was 80 and the corresponding TE values were in the 50–4000 ms range. The analysis of the obtained data was based on the linear dependence of the relaxation rates $\frac{1}{T_{\mathrm{iObs}}}$ of the series of dilution on the concentration of gadolinium incorporated into nanocapsule layers using the formula:

2 $$\frac{1}{T_{\mathrm{iObs}}}=\frac{1}{T_{\mathrm{iS}}}+r_{i}•C;\;i=\left(1,2\right)$$

where $\frac{1}{T_{\mathrm{iS}}}$ is a diamagnetic term, corresponding to the relaxation rate of the solvent without the contrast agent, and *r*_*i*_•*C* is a paramagnetic term, which describes the relaxation rate enhancement due to the paramagnetic Gd presence. *T_i_* (*i* = 1,2) means spin–lattice and spin–spin nuclear magnetic relaxation times, respectively. The paramagnetic contribution is proportional to the concentration *C* of gadolinium and the specific proton relaxivity, *r*_*i*_, which characterizes the efficiency of the relaxation rate enhancement by the given paramagnetic center. In order to directly compare the relative relaxation properties of one- and two-layer PLL-Gd-doped nanocapsules with the nondoped ones, the linear dependence of the relaxation rates on the nanocapsule (instead of Gd) concentration was analyzed as well, resulting in the *r*_iNC_ coefficient (see Table 3). Analysis of the measured signal intensities was accomplished using Paravision 6.1 and MATLAB (MathWorks) software.

#### 2.2.9 Cell Culture and Treatment

##### 2.2.9.1 SH-SY5Y Cell Cultures

SH-SY5Y cells (passages 5–25) were cultured in DMEM with high glucose containing 10% (v/v) heat-inactivated FBS and 1% (v/v) penicillin/streptomycin mixture^47^ and incubated under standard conditions (37 °C, 5% CO_2_, >95% humidity). Cells were grown in plastic T75 culture flasks to reach 80% confluence and then were subcultivated using 0.05% trypsin/EDTA solution. For experiments on undifferentiated SH-SY5Y cells, after trypsinization, they were counted manually in a Bürker chamber, seeded into 96-well plates with density 5 × 10^4^ cells/well in 100–200 μL of supplemented DMEM and incubated for 24–48 h to ensure optimal cell growth. One day before biological experiments, the culture medium was exchanged to fresh serum-free DMEM containing 1% (v/v) antibiotics and 1% (v/v) N-2 supplement.

##### 2.2.9.2 SH-SY5Y Cell Treatment

SH-SY5Y cells were treated with appropriately diluted nanocarriers (10 μL per well, final dilutions in the culture medium 10, 20, 40, 80, 100, 200, and 500×, respectively, depending on the type of sample) and incubated for 24 h under standard conditions to validate their putative cytotoxicity. In all described experiments, NCs were freshly diluted in 15 mM NaCl solution and control cells were supplemented with a vehicle (10 μL of 15 mM NaCl solution). Moreover, selected wells with cells lysed with 1% (v/v) Triton X-100 were used as a control for the utmost cell membrane damage.

##### 2.2.9.3 Primary Neuronal Cell Cultures

The forebrain tissue for the preparation of primary neuronal cortical cell cultures was sourced from 15/16-day embryos which were taken from pregnant CD1 mice (Charles River Laboratories, Sulzfeld, Germany). Primary neuronal cell cultures were prepared according to the procedure described previously^48^ and this protocol is in line with the European Union (Directive 2010/63/EU, amended by Regulation (EU) 2019.1010) guidelines on the ethical use of animals. The cells isolated from the trypsynized brain tissue (0.1% trypsin in PBS w/o Ca^2+^/Mg^2+^, 20 min at room temperature) were manually counted (Bürker chamber) and seeded at density 6 × 10^4^ cells per well in poly-l-ornithine (0.05 mg/mL)-covered 96-well plates. The cells were maintained in the neuronal cell culture medium (Neurobasal A medium supplemented with 2 mM glutamine, 0.4% B27 supplement, 0.06 μg/mL penicillin, and 0.1 μg/mL streptomycin) at 37 °C in a humidified atmosphere containing 5% CO_2_. For the first 2 days, 5% (v/v) FBS was added to the cell culture medium to improve cell survival. The primary neuronal cells were cultivated for 8 days prior to experimentation with medium exchange every 2 days.

##### 2.2.9.4 Cell Treatment of Primary Neuronal Cell Cultures

The primary neuronal cell cultures were treated for 24 h with 10% (v/v) of various dilutions (10, 20, and 40×) of tested NCs. The control cells were supplemented with a vehicle (10% (v/v) sterilized 15 mM NaCl solution).

##### 2.2.9.5 hCMEC/D3 Cell Culture

The brain microvascular endothelial hCMEC/D3 cell line was grown in 75 cm^2^ flasks in EBM-2 medium complemented with human basic fibroblast growth factor (bFGF, 1 ng/mL, dissolved in DPBS and diluted in pure EBM-2 according to the manufacturer’s protocol), ascorbic acid (5 μg/mL, dissolved in water), hydrocortisone (500 ng/mL, dissolved in ethanol), 1% (v/v) chemically defined lipid concentrate, 1% (v/v) HEPES, 1% (v/v) penicillin/streptomycin mixture (100 units/mL penicillin and 100 μg/mL streptomycin), and 5% (v/v) heat-inactivated FBS. Human bFGF was added to the prepared medium right before cultivation, medium change, or seeding of cells. Cell culture flasks were precoated with rat collagen type I (final protein concentration: 150 μg/mL) at least 1 h prior to use and placed in the incubator. Before seeding cells, the collagen was discarded, and flasks were washed twice with DPBS. Subsequently, cells were cultured in a humidified atmosphere with 5% CO_2_ at 37 °C to reach confluence. The culture medium was discarded and replaced with a fresh medium every 2 days. After reaching 80% confluency, hCMEC/D3 cells were detached using 0.25% trypsin/EDTA solution (15–20 min, 37 °C), collected by centrifugation (8 min, 900 rpm), resuspended in the fresh medium, and counted manually (Bürker chamber). To ensure optimal growth conditions, the cells were plated in density 4.5 × 10^4^ cells per well into appropriate 24-well plates (Corning) on rat collagen type I-coated Transwell filters (0.15 mg/mL, 1 h prior to use in 37 °C and after double washing the inserts with DPBS). Cells were incubated in a controlled environment (37 °C, 5% CO_2_, 95% humidity) until a confluent monolayer was formed. The culture medium was superseded by the fresh medium every 2 days and before the treatment. In order to avoid loss of BBB properties, the hCMEC/D3 cells were used between passages 27 and 35 according to the manufacturer’s recommendations.

#### 2.2.10 hCMEC/D3 Cell Treatment

The apical chamber (Transwell inserts) of each well was loaded with rhodamine-labeled nanocarriers (75 or 150 μL of AOT 1xROD or PCL 1xROD) and the appropriate volume of EBM-2 medium was refilled up to 750 μL. The basolateral chamber (well beneath the insert) contained 750 μL of FluoroBrite DMEM, which guarantees unbothered measurements of fluorescence. The FluoroBrite DMEM does not consist of components showing the fluorescence signal inter alia phenol red and therefore seems to be adequate for those kinds of experiments. Afterward, 50 μL portions of the medium from basolateral chambers were collected at consecutive time points and transferred to a 96-well black plate. The volume in the wells was refilled with 50 μL of FluoroBrite DMEM. Similarly, the wells beneath the inserts were replenished with the fresh medium at each time point. Between the measurements, the plates were incubated at 37 °C under standard conditions. The fluorescence signal referring to the crossing ability through the BBB was evaluated at 558 and 586 nm (the excitation and emission wavelengths, respectively) using a multiwell plate reader Infinite M200 PRO (Tecan Gmbh, Austria) after various times from the exposure to NCs. Furthermore, control experiments were carried out to measure the fluorescence from cells that were not exposed to NCs or to check the transport of NCs through empty inserts (only with the collagen coating). Finally, all recorded fluorescence values were adjusted by subtracting the baseline value from the FluoroBrite DMEM medium.

#### 2.2.11 Transepithelial Electrical Resistance (TEER) Measurements

In order to determine the permeability of plated hCMEC/D3 cells and to confirm the presence of a tight, confluent monolayer, transepithelial electrical resistance (TEER, in Ω·cm^2^) was measured on 8 following days using the Millicell ERS-2 electrical resistance system (epithelial volt–ohm meter, Merck Millipore, Burlington, MA). TEER is a standard tool and a straightforward indicator for judging the integrity and function of epithelial cells *in vitro*. It is particularly useful due to the fact that it allows assessment of barrier tissue development and monitoring of the dynamic changes in a nondestructive and time-dependent way. To evaluate the validated resistance, the electrode was equilibrated according to the manufacturer’s recommendations and sterilized in 70% ethanol for 15 min before the experiments. The possible influence of temperature was minimized by performing measurements after at least 5 min of incubation of culture plates in ambient conditions. Collagen-coated blank Transwell inserts (without cells) were considered a control in the TEER measurements and were subtracted from the final values. The resistance was assessed every 24 h, and the TEER was calculated by multiplication of the Transwell inserts’ area with the obtained resistance value. The model of the BBB is completely prepared for further experiments when a tight junction appears and the resistance reaches the highest value.

#### 2.2.12 Cellular Uptake

To evaluate the cellular uptake of NCs, SH-SY5Y cells were seeded at density 1.5 × 10^5^ cells per well in 24-well plates in a cell culture medium containing 10% (v/v) FBS and 1% (v/v) penicillin/streptomycin solution. One day after cell seeding, the culture medium in wells was replaced with an experimental medium (DMEM, 1% (v/v) penicillin/streptomycin solution, and 1% (v/v) N-2 supplement). The next day after medium exchange, 10% (v/v) tested NCs (three different batches in each type, dilution 40×) were added to cells for 30 min and 1, 3, 6, and 24 h. Control cells were supplemented with a vehicle (10% (v/v) sterilized 15 mM NaCl solution). After cell treatment and washing the cells with prewarmed FluoroBrite DMEM, they were collected on ice into 1.5 mL tubes, centrifuged (4 °C, 180*g*, 5 min), and resuspended in 200 μL of cold DPBS (without calcium and magnesium). One ×10^4^ cells were analyzed using a BD FACSAria Fusion cell sorter and FACSDiva 9.2 software (BD Biosciences, San Jose, CA) in the fluorescence channel for PE-A (red fluorescence). Data are presented as a percentage of rhodamine-positive cells and the mean rhodamine fluorescent intensity (±SEM) established from 3 independent experiments.

#### 2.2.13 MTT Cell Viability Assay

For the assessment of the cell viability of primary neuronal cell cultures, 3-[4,5-dimethylthiazol-2-yl]-2,5-diphenyltetrazolium bromide (MTT) assay was used as described previously.^48^ The data were normalized to the control (vehicle-treated cells, 100%) and are presented as a mean ± SEM from 3 independent experiments with 3–5 replicates each.

#### 2.2.14 WST-1 Cell Viability Assay

The viability of the SH-SY5Y cells exposed to different concentrations of NCs was estimated by biochemical WST-1 assay as described in detail previously.^49^ The data were normalized to the control (vehicle-treated cells, 100%) and are shown as a percentage of control ± SEM from at least 4 independent experiments with 3–5 replicates each.

#### 2.2.15 LDH Release Assay

In order to estimate the potential cytotoxicity of studied NCs, the level of lactate dehydrogenase (LDH) release from treated cells to the culture medium was estimated as a credible marker of cell death according the procedure described in detail in our previous study.^49^ The data were normalized to the control (vehicle-treated cells, 100%) and are presented as a percentage of control ± SEM from at least 3 independent experiments with 3–5 replicates each.

#### 2.2.16 Statistical Analysis

To evaluate the significance between treatments, the data were analyzed with Statistica 13.3 software (StatSoft Inc., Tulsa, OK) with assumed *p* < 0.05. The one-way or two-way analysis of variance (ANOVA) and *posthoc* Duncan’s test were employed to assess the statistical significance between various experimental groups.

## 3 Results and Discussion

### 3.1 Synthesis of Polymeric-Based NCs of Selected Neuroprotective Drugs (Cyclosporin A (CsA) and Tacrolimus (FK506))

The neuroprotectant-loaded polymeric-based theranostic NCs were synthesized according to our previous reports.^22,36,50^ The method is based on a nanoemulsion template with further functionalization by the layer-by-layer approach. The drug-loaded nanocores were prepared using the spontaneous emulsification–solvent evaporation method, in the case of the solid core, additionally enhanced by the ouzo effect.^51^ The procedure of preparation was as follows: 0.1 mL of the oil phase or the oil phase prior mixed with absolute ethanol (1:100) was added to 200 mL of poly-l-lysine solution in 15 mM NaCl. The process was performed at room temperature and under continuous mixing using a magnetic stirrer at 300–500 rpm. Once the phases were mixed, the emulsification process occurred, and a nanoemulsion stabilized by the interfacial complex of AOT/PLL was formed. The concentration of PLL needed to form the stable complex and minimize the presence of free molecules of polyelectrolytes was previously established and was 0.2 g/L.^36^ Then, after evaporation of the organic solvent (chloroform), drug-loaded nanocarriers were formed. The concentrations of selected drugs CsA and FK506 were optimized to achieve as high as possible DEE. The concentrations of drugs in the oil phase varied from 3.1 to 100 g/L, and the optimal concentration was the highest where no precipitation after the formation of the nanoemulsion was observed, i.e., 50 g/L. The drug entrapment efficacy (DEE) in both nanocores was determined by the centrifugation and UV–vis spectroscopy method. Namely, the final suspensions of drug-loaded nanocores were centrifuged with the spin rate of 10,000 rpm at 25 °C for 60 min, and the UV–vis spectroscopy analysis of the filtrate did not reveal the presence of free drug molecules. Moreover, selected drugs are not soluble in water; therefore, we assumed 100% efficacy of encapsulation. The calculated concentration of CsA and FK506 in the drug-loaded nanocore suspensions was 25 mg/L for both types of NCs, which corresponds to 21 and 31 μM, respectively. The empty ones as the corresponding references were prepared similarly, excluding drug addition in the above procedure. The mean size of obtained drug-loaded nanocores and their corresponding references ranged from 90 to 110 nm, with a polydispersity index of less than 0.15 (Figure 2). The nanoparticle concentration determined by NTA was ∼1 × 10^12^ nanoparticles/mL. The zeta potential of the drug-loaded and empty nanocores was in the range of +60 to +75 mV, depending on the type of nanocore. The value was high enough to prevent the aggregation process by providing electrostatic stabilization; moreover, such a high potential was a good basis for building the multifunctional polyelectrolyte shell for MRI or OI using the LbL assembly technique.

**Figure 2:**
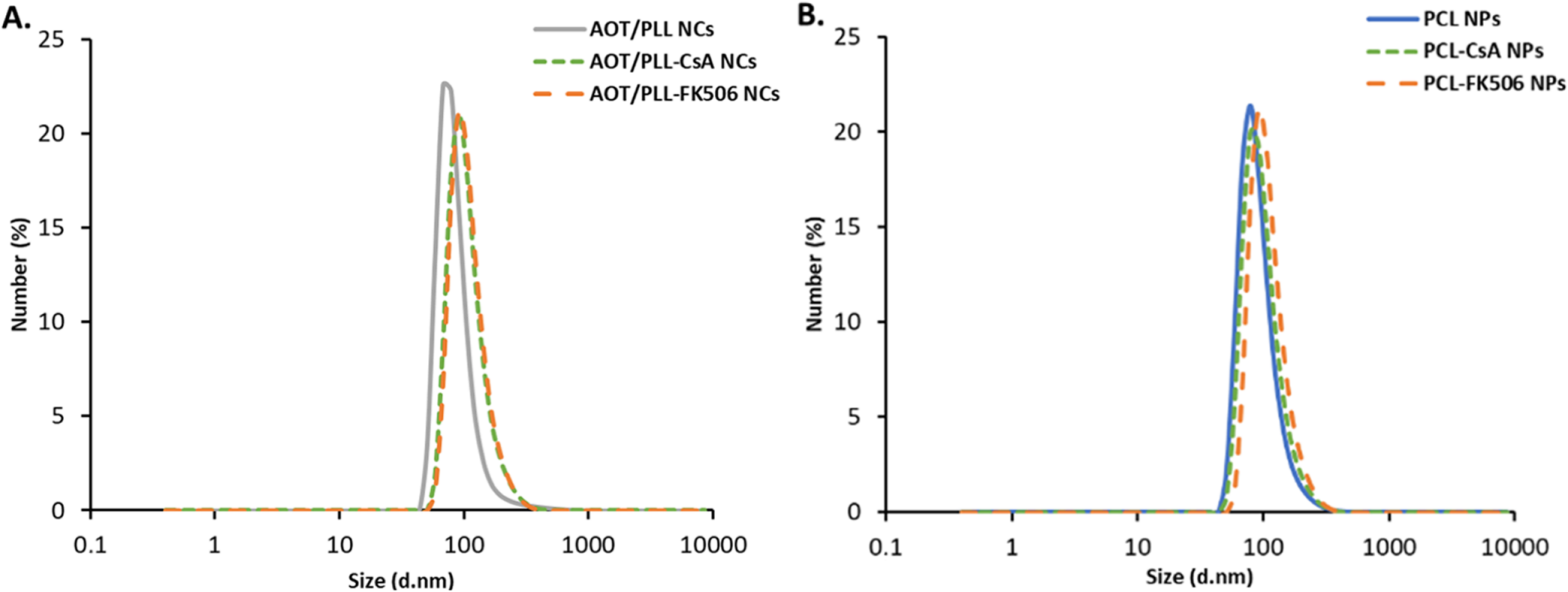
Mean size of the drug-loaded (A) AOT nanocores and (B) PCL nanocores using Dynamic Light Scattering (DLS), with corresponding references.

### 3.2 Polymeric-Based NC Functionalization for Magnetic Resonance Imaging and Optical Imaging

Functionalization of polymeric-based nanocarriers for imaging was achieved by multilayer shell formation using sequential adsorption of charged molecules (the layer-by-layer method). To form the multilayer shell, the following molecules were selected: positively charged polyelectrolytes: poly-l-lysine (PLL); gadolinium-labeled poly-l-lysine, (PLL-Gd); and rhodamine-labeled poly-l-lysine (PLL-ROD) and negatively charged polyelectrolytes: poly-l-glutamic acid (PGA) and pegylated poly-l-glutamic acid (PGA-g-PEG). Here, two types of multifunctional polyelectrolyte shells with a different number of layers were built up, one dedicated to MRI with PLL-Gd as a contrast agent and the second with PLL-ROD for OI. The formation of multilayer shells was confirmed by changes in the zeta potential value after each adsorption step (Figure 3a). An external layer of all nanocarrier shells was created with PGA-g-PEG to form pegylated NCs with a largely reduced zeta potential. The pegylation is aimed primarily to provide steric stabilization of NCs under physiological conditions. To facilitate delivery to the central nervous system, neutral nanocarriers must exhibit prolonged circulation by evading binding to immune cells and proteins within the bloodstream. This extended circulation is paramount for effective CNS targeting. Figure 3b presents examples of the size distributions of prepared polymeric-based theranostic NCs. The mean sizes of NCs obtained by DLS were below 250 nm, which was additionally confirmed by cryo-SEM analysis. The example of cryo-SEM micrographs is demonstrated in Figure 3c. Table 2 summarizes the abbreviations, detailed composition, and characteristics (size, PDI, and zeta potential) of prepared NCs. The stability studies showed that the NCs are stable for at least 2 weeks, which was confirmed by the time dependence measurements of the size distribution and zeta potential. All prepared samples possessed the corresponding references, i.e., composed with unlabeled PLL.

**Table 2:** Abbreviations, Detailed Composition, and Characteristics (Size, PDI, and Zeta Potential) of Prepared Nanocarriers

	**sample code**	**size**[nm]	**PDI**	**zeta potential**[mV]
	core type			
	shell composition			
**MRI Gd-labeled NCs**	**AOT 1xGd**	**160**	**0.24**	**–4.6**
	liquid			
	PLL/PGA/PLL-Gd/PGA-g-PEG			
	**AOT 2xGd**	**225**	**0.13**	**–0.4**
	liquid			
	PLL/PGA/PLL-Gd/PGA/PLL-Gd/PGA-g-PEG			
	**PCL 1xGd**	**106**	**0.28**	**–3.4**
	solid			
	PLL/PGA/PLL-Gd/PGA-g-PEG			
	**PCL 2xGd**	**122**	**0.17**	**+1.4**
	solid			
	PLL/PGA/PLL-Gd/PGA/PLL-Gd/PGA-g-PEG			
**OI ROD-labeled NCs**	**AOT 1xROD**	**106**	**0.18**	**+0.5**
	liquid			
	PLL/PGA/PLL-ROD/PGA-g-PEG			
	**AOT 2xROD**	**164**	**0.26**	**–6.0**
	liquid			
	PLL/PGA/PLL-ROD/PGA/PLL-ROD/PGA-g-PEG			
	**PCL 1xROD**	**106**	**0.30**	**–5.3**
	solid			
	PLL/PGA/PLL-ROD/PGA-g-PEG			
	**PCL 2xROD**	**141**	**0.20**	**–1.0**
	solid			
	PLL/PGA/PLL-ROD/PGA/PLL-ROD/PGA-g-PEG			

**Figure 3:**
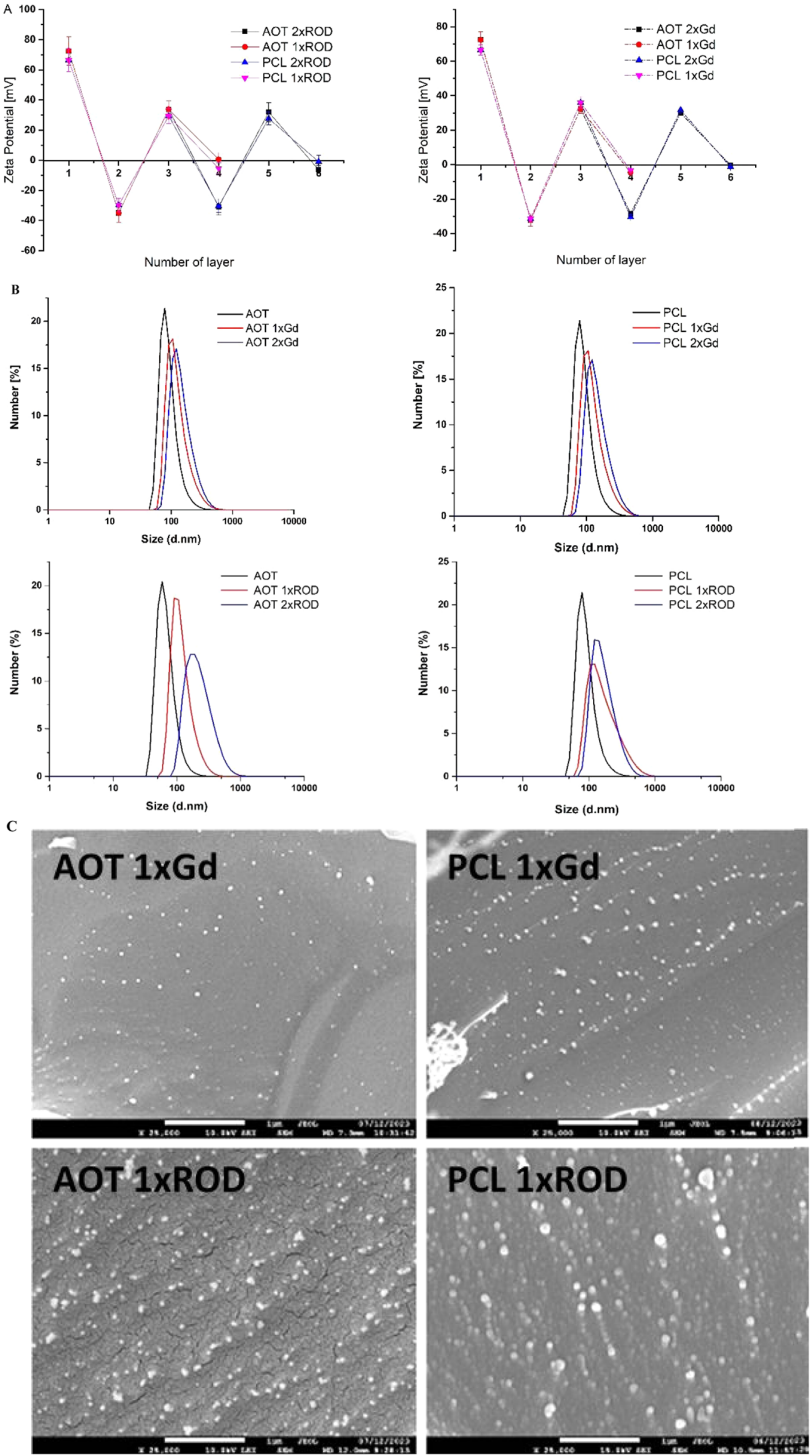
(A) Changes in the zeta potential value after the formation of each layer for OI and MRI, as confirmation of multilayer shell formation (presented values for empty NCs). (B) Example of size distributions measured by DLS (AOT and PCL carriers with the functional shell for MRI and OI) and (C) cryo-SEM images of selected polymeric-based theranostic nanocarriers (both types: AOT and PCL) with the functional shell for MRI and OI, containing one labeled layer (AOT 1xROD, AOT 1xGd, PCL 1xROD, PCL 1xGd). Scale bar: 1 μm.

### 3.3 Optical Imaging: Fluorescence Spectroscopy, Microscopy, and IVIS

Among the many fluorescent probes developed, rhodamine dyes are widely used in biotechnology as fluorescent markers or for detecting biomolecules, owing to their excellent optical and physical properties.^52^ Accordingly, rhodamine was selected for labeling polymeric-based nanocarriers. The rhodamine-labeled polycation PLL-ROD was used to form polymeric NCs for optical imaging. The characteristic emission band of rhodamine at ∼580 nm could be observed, as demonstrated in Figure 4A. Moreover, formed nanocarriers were incorporated into agarose gel and visualized by confocal microscopy. The examples of the image are also illustrated in Figure 4B.

**Figure 4:**
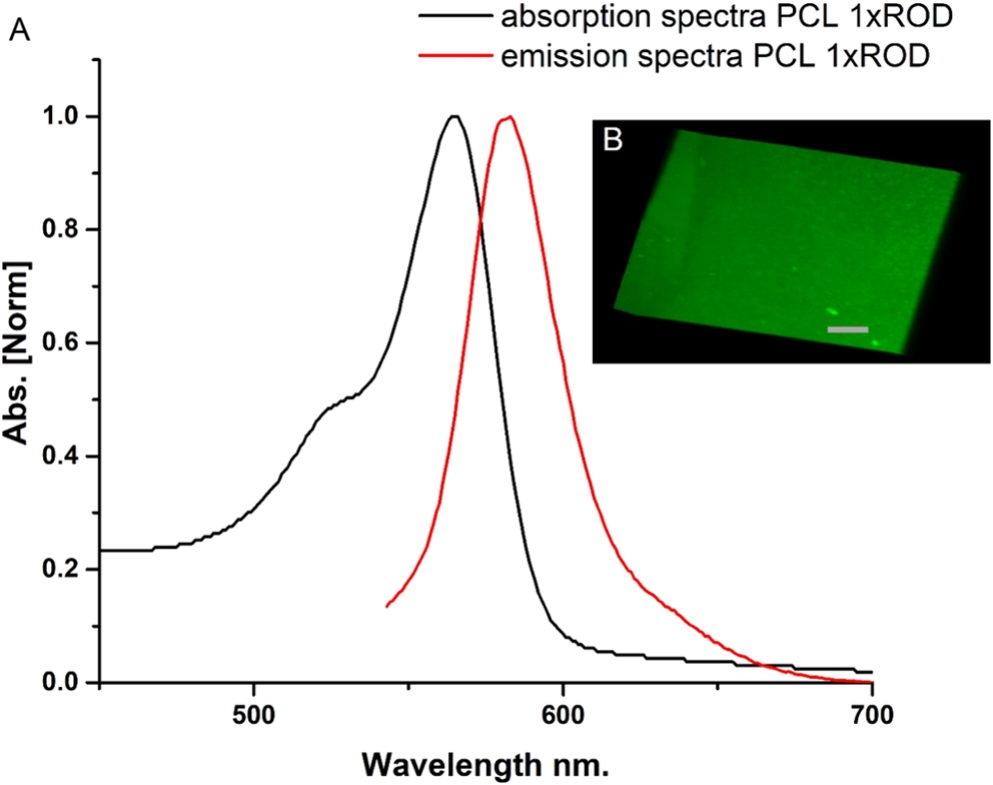
(A) Absorption and emission spectra of fluorescently labeled NCs and (B) confocal image of PCL 1xROD nanocarriers incorporated into agarose gel. Scale bar: 40 μm.

The presented results prove that proposed fluorescently labeled NCs have beneficial properties for bioimaging, e.g., evaluation of the biodistribution. With sufficient accumulation in the site of action, our NCs can serve as both transport devices for therapeutic cargo and imaging compounds for distribution assessment.

### 3.4 Magnetic Resonance Relaxometry and Imaging of Developed Polymeric-Based Theranostic NCs

The samples containing polymeric-based NCs of solid (PCL) and liquid (AOT) cores and with the functional shell containing one or two PLL-Gd layers were investigated. The representative example illustrating *T*_1_ and *T*_2_weighting in the set of dilutions of PCL samples with incorporated two layers of PLL-Gd is presented in Figure 5.

**Figure 5:**
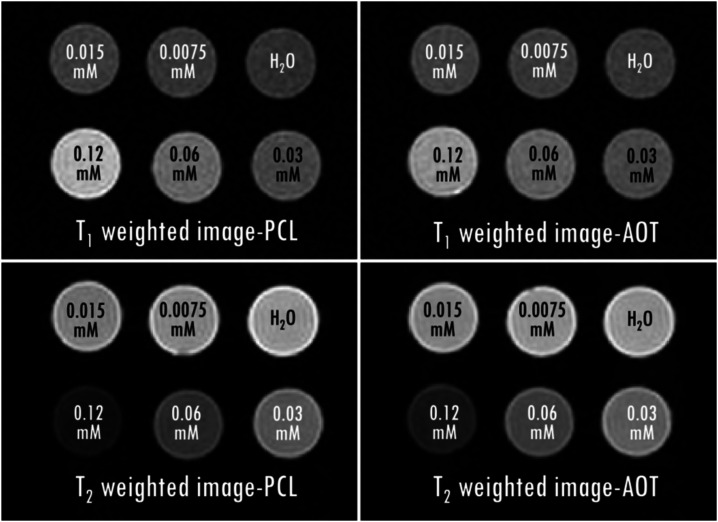
Contrasting properties of PCL and AOT NCs with double PLL-Gd layers are illustrated in *T*_1_- and *T*_2‑_weighted MR images. In the *T*_1_-weighted image (acquired using a spin–echo RARE sequence with the following parameters: TE 7 ms, TR 120 ms, MTX 128 × 128), the brightest sample corresponds to the highest concentration of the contrast agent at 0.12 mM, which exhibits the shortest *T*_1_ relaxation time. In the *T*_2_-weighted image (acquired using a spin–echo MSME sequence with the following parameters: TE 200 ms, TR 15,000 ms, MTX 128 × 128), the same sample appears the darkest due to its shortest *T*_2_ relaxation time.

All of the studied nanocarriers exhibited decent MRI properties. In particular, the molar relaxivities *r*_1_ exhibit values between 2.53 and 3.71 mM^–1^s^–1^ (see Table 3), similar to contrast agents which are commercially used. The molar relaxivities of *r*_2_ were between 11.9 and 22.8 mM^–1^s^–1^. This introduces some *T*_2_ contrast, especially if the appropriate pulse sequence parameters are applied (see Figure 5). The molar relaxivities of the single- and double-layer PLL-Gd NCs (calculated with respect to the Gd concentration) were in a similar range. Analysis with respect to the concentration of the whole NCs (Table 4) indicates the multifunctionality of the investigated compounds. Namely, increasing the number of PLL-Gd layers, and thus the gadolinium concentration per NC, enhances the resulting contrasting effect. On the other hand, a similar image contrast enhancement can be achieved for NCs with only a single layer of PLL-Gd, if the total concentration of NCs is increased. In the second case, the higher concentration of therapeutic agents can be delivered. The presented results prove that proposed NCs should have beneficial contrasting properties for the evaluation of the distribution of theranostic NCs *in vivo*. The *r*_1_ molar relaxivity values, similar to clinically used contrast agents, suggest that with sufficient accumulation in the site of action, the prepared NCs can serve as both transport devices for therapeutic cargo and MRI compounds for distribution assessment.

**Table 3:** *r*_1_ and *r*_2_ Results for Samples with PCL and AOT/PLL with 1 or 2 Layers of PLL-Gd^a^

	*r*_1_ [s^–1^mM^–1^]		*r*_2_ [ s^–1^mM^–1^]	
NCs	1 layer of PLL-Gd	2 layers of PLL-Gd	1 layer of PLL-Gd	2 layers of PLL-Gd
AOT/PLL	3.711 ± 0.050	2.534 ± 0.030	11.9 ± 1.4	15.49 ± 0.47
PCL	3.64 ± 0.20	3.285 ± 0.031	22.8 ± 1.9	16.82 ± 0.28

a *r*_1_ and *r*_2_ are calculated relative to the gadolinium concentration.

**Table 4:** Comparison of *r*_1NP_ and *r*_2NP_ Results for Samples with PCL and AOT/PLL with 1 or 2 Layers of PLL-Gd with the Corresponding Samples without Gd (ref)^a^

	*r*_1NP_ [s^–1^mM^–1^]		*r*_2NP_ [s^–1^mM^–1^]	
NCs	1 layer of Gd	2 layers Gd	1 layer of Gd	2 layers Gd
AOT/PLL	0.204 ± 0.003	0.566 ± 0.007	0.65 ± 0.07	3.46 ± 0.11
PCL	0.200 ± 0.11	0.733 ± 0.007	1.25 ± 0.11	3.75 ± 0.06
AOT/PLL ref	0.024 ± 0.005	0.019 ± 0.005	0.16 ± 0.12	0.34 ± 0.04
PCL ref	0.024 ± 0.005	0.021 ± 0.011	0.71 ± 0.18	0.49 ± 0.05

a *r*_1NP_ and *r*_2NP_ are calculated relative to the nanoparticle concentration.

### 3.5 Biosafety of Polymeric-Based Theranostic NCs

#### 3.5.1 Effect of Developed Polymeric-Based Theranostic NCs in SH-SY5Y Cell Cultures

In order to analyze the biosafety of empty NCs, toxicity tests using the human neuroblastoma SH-SY5Y cell line were performed. First, the LDH release levels for AOT 1xGd, PCL 1xGd, AOT 1xROD, and PCL 2xROD NCs and the corresponding references were evaluated (Figure 6A). Only the highest applied NC concentrations exerted a significant cytotoxic effect against the cells. In contrast, NCs in the dilution 20× or bigger did not evoke any detrimental effect on cells compared to the control group treated with the vehicle. The exception was AOT 1xGd, in which 20× dilution was slightly more toxic than the same concentration of AOT without Gd. The data from the LDH release were confirmed by the WST-1 assay performed on cells treated with NCs for 24 h (Figure 6B), where 20× dilutions were considered safe. To determine if the increasing amount of the gadolinium complex incorporated in the shell of nanocarriers influenced its biosafety on SH-SY5Y cultures, the potentially harmful effects of AOT 2xGd and PCL 2xGd were explored. The obtained results clearly indicate that only for the highest concentrations employed, a significant increase of the LDH level was noticed (Figure 7A). Moreover, the viability of cells was slightly reduced, as shown by the WST-1 assay (Figure 7B). No detectable toxicity and decrease in viability for dilutions higher than 20× were found. This observation is in good agreement with previous results for NCs with four external layers. It is worth noting that a comparison of two types of NC cores (AOT and PCL) shows no meaningful difference between their cytotoxic potentials, at least in SH-SY5Y cells. Therefore, both types of NCs are promising candidates as neuroprotective drug NCs.

**Figure 6:**
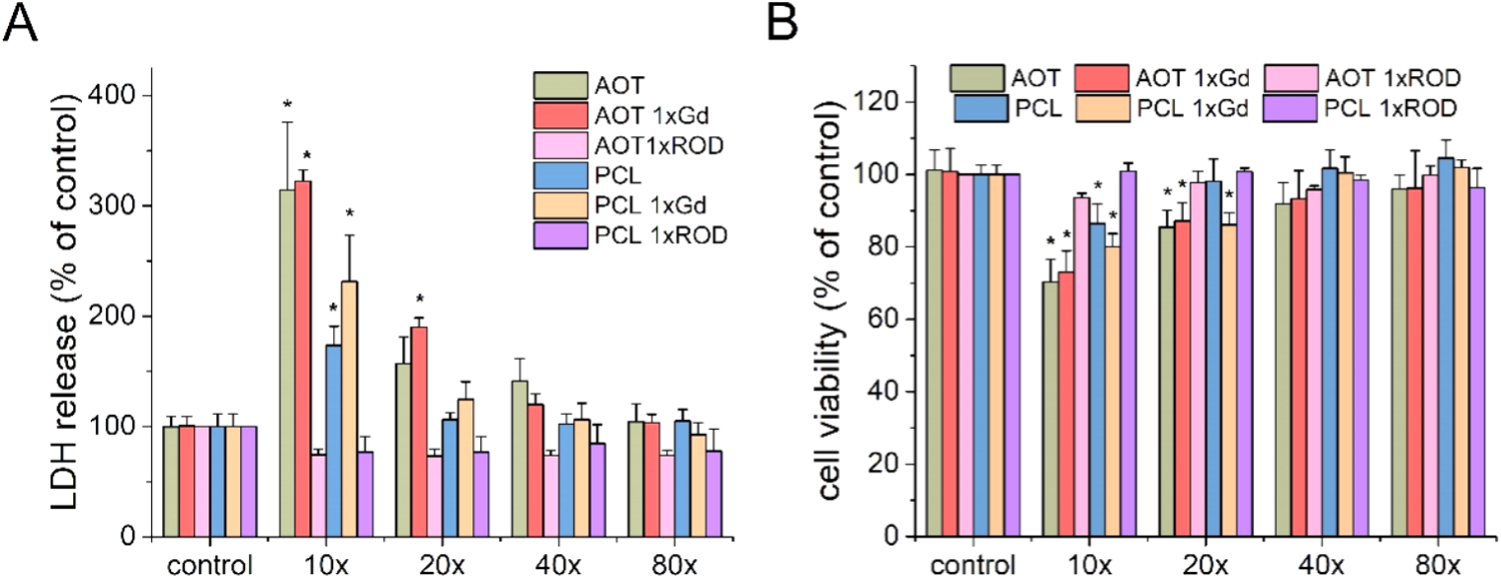
Effect of AOT 1xGd, PCL 1xGd, AOT 1xROD, and PCL 1xROD NCs and the corresponding references at varying dilutions on the (A) LDH release and (B) cell viability on SH-SY5Y cells. The data were recalculated to the control (vehicle-treated cells) and expressed as the mean (in percent) ± SEM from 4–6 independent experiments with 3 replicates. *p < 0.05 vs control.

**Figure 7:**
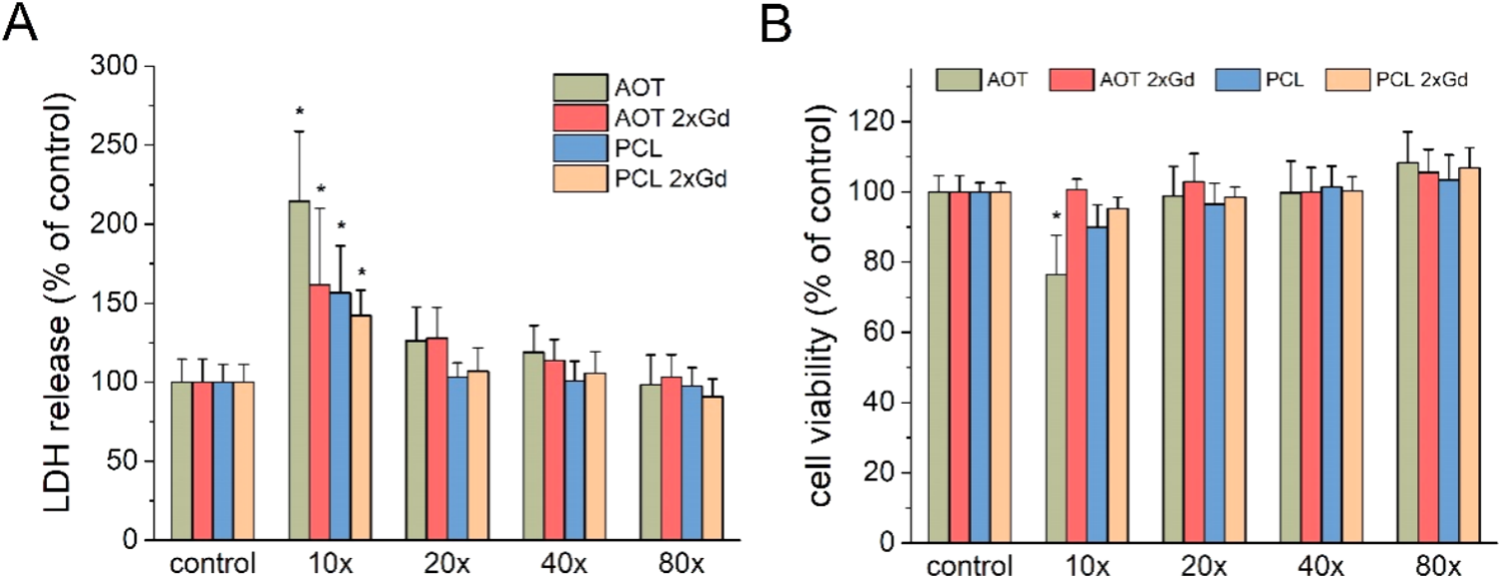
Effect of AOT 2xGd, PCL 2xGd NCs, and the corresponding references at varying dilutions on the (A) LDH release and (B) cell viability on SH-SY5Y cells. The data were recalculated to the control (vehicle-treated cells) and expressed as the mean (in percent) ± SEM from 4–6 independent experiments with 3 replicates. *p < 0.05 vs control.

#### 3.5.2 Effect of Polymeric-Based Theranostic NCs in Primary Neuronal Cell Cultures

The biosafety of developed NCs in primary neuronal cell cultures was also evaluated. Twenty-four hours of treatment with AOT NCs (reference sample) at dilution 10× evoked a significant increase in LDH release but did not affect the cell viability. No detrimental effect on cells of AOT NCs at dilutions of 20× and 40× was observed. AOT 1xGd at dilutions 10× and 20× evoked a significant decrease in cell viability and an increase in LDH release. Two-way ANOVA revealed a significantly higher cell-damaging effect of AOT 1xGd NCs vs AOT ones at dilution 20× in both biochemical assays; however, dilution 40× of AOT 1xGd was safe for primary neuronal cell cultures (Figure 8A,B). The comparison of two types of NC cores containing the PLL-Gd layer (AOT 1xGd and PCL 1xGd) with each other shows no meaningful difference in their cytotoxic potential, which was observed for dilutions 10× and 20× (Figure 8C,D). Therefore, both types of NCs at dilution 40× could be regarded as safe for primary neuronal cell cultures for further investigations on the neuroprotective potency of NCs containing drugs.

**Figure 8:**
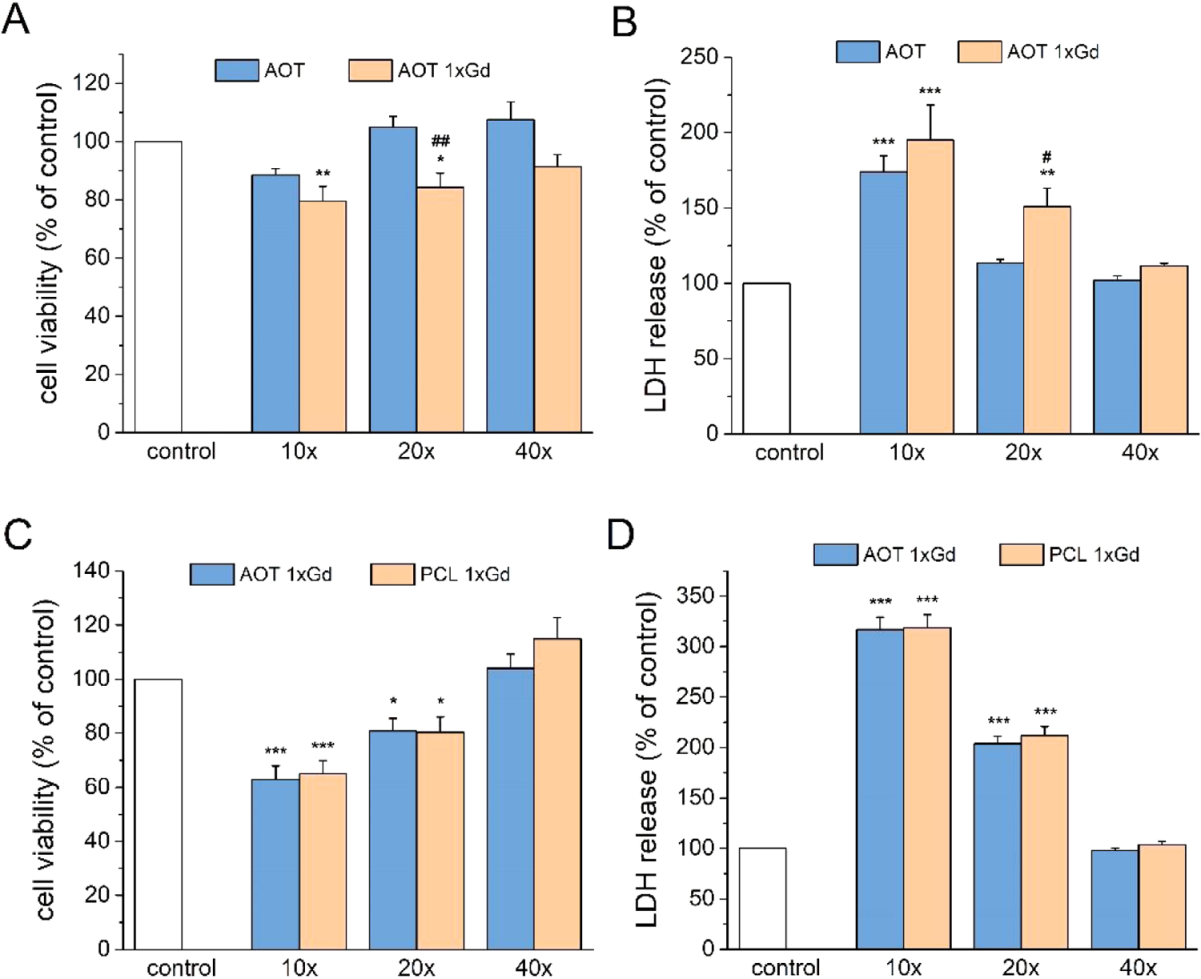
Effect of AOT, AOT 1xGd, and PCL 1xGd NCs at different dilutions on the (A, C) cell viability and (B, D) LDH release in primary neuronal cell cultures. The data were normalized to the control (vehicle-treated cells) and expressed as the mean (in percent) ± SEM from 3 independent experiments with 3–5 replicates. *p < 0.05, **p < 0.01, and ***p < 0.001 vs control; #*p* < 0.05 and ##*p* < 0.01 AOT 1xGd vs AOT-treated cells.

#### 3.5.3 Cellular Uptake of Rhodamine-Labeled NCs in SH-SY5Y Cells

The SH-SY5Y cells were used as a model to investigate the cellular uptake of rhodamine-labeled NCs. The NCs tested at safe dilution (40×) were taken by cells in a time-dependent manner, where we observed ∼20% of rhodamine-positive cells after 30 min of treatment, ∼30% after 1 h, ∼60% after 3 h, ∼70% after 6 h, and ∼80% after 24 h (Figure 9). A significant increase in cellular uptake was detected between time points 30 vs 0 min (vehicle-treated cells) and 3 vs 1 h for both tested types of NCs. No differences in the number of rhodamine-positive cells between both types of NC cores (AOT and PCL) were observed (Figure 9A). The increase in mean rhodamine fluorescence in cells is evidence of the cellular accumulation of NCs. A significant increase in this parameter in cells treated for 3, 6, and 24 h when compared to vehicle-treated cells was shown (Figure 9B). Moreover, meaningful differences in mean rhodamine fluorescence for NCs between time points 6 and 24 h were noticed. By two-way ANOVA analysis, we did not observe any differences in mean rhodamine fluorescence between both types of nanocarrier cores (AOT and PCL) at any tested time point.

**Figure 9:**
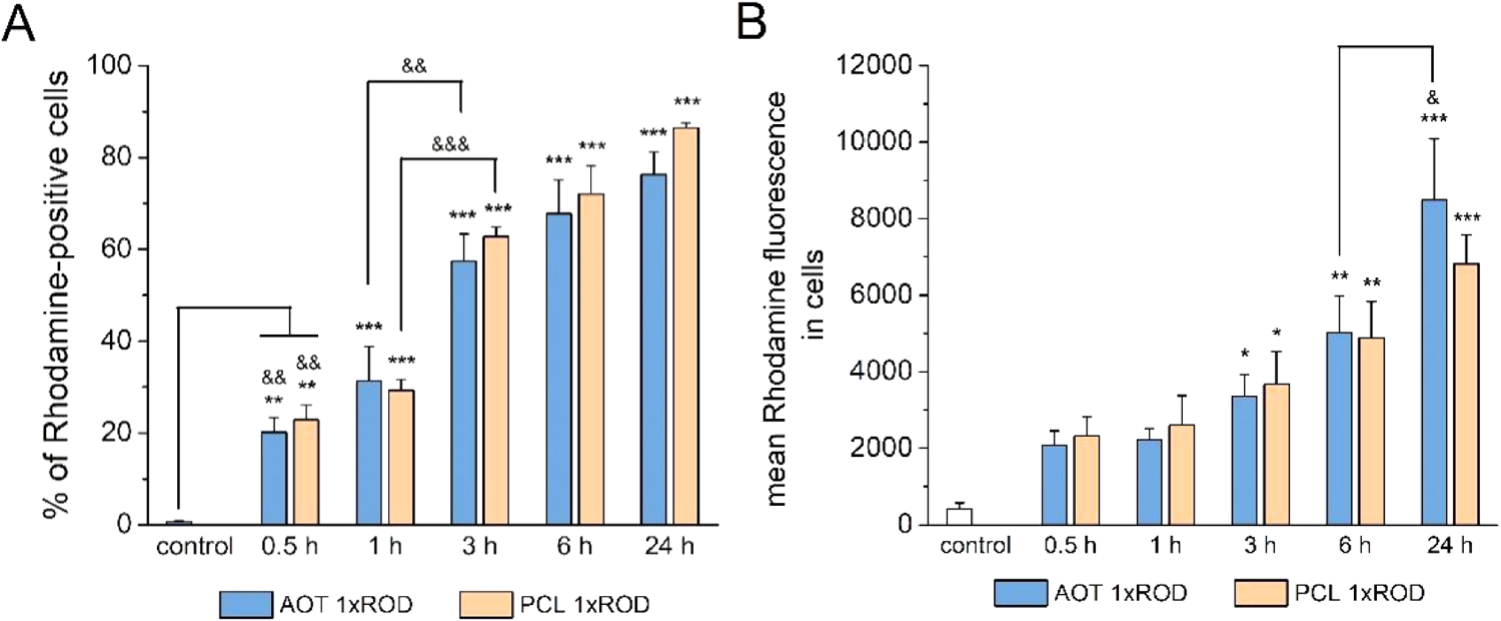
Cellular uptake of AOT 1xROD and PCL 1xROD by SH-SY5Y cells. The data are presented as (A) a percentage of rhodamine-positive cells and (B) the mean rhodamine fluorescence intensity ± SEM from 3 independent experiments. *p < 0.05, **p < 0.01, and ***p < 0.001 vs control (vehicle-treated cells); &*p* < 0.05, &&*p* < 0.01, and &&&*p* < 0.001 vs indicated time points.

#### 3.5.4 Transfer through the BBB Model

The results obtained for hCMEC/D3 cells revealed that TEER values gradually increase up to the fifth day, when the resistance reaches the maximum (Figure 10). This indicates that 5 days after seeding, cells are most tightly packed and thus ready for further experiments.

**Figure 10:**
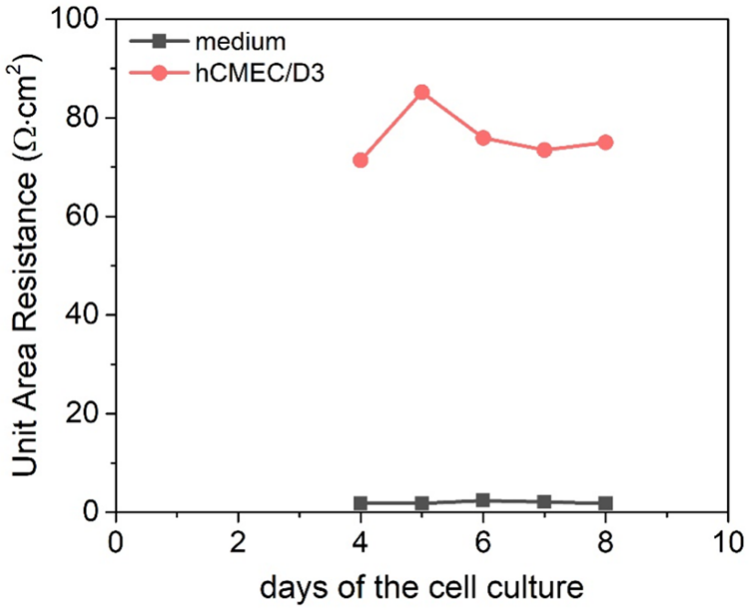
Transelectrical resistance measurements on the hCMEC/D3 monolayer over a period of 8 days.

To determine the kinetics of NC transfer through the BBB model, we investigated the permeability of the hCMEC/D3 cell monolayer for AOT 1xROD and PCL 1xROD NCs using fluorescence intensity measurements. We observed that both types of NCs can cross this cellular BBB model in a time-dependent manner (Figure 11).

**Figure 11:**
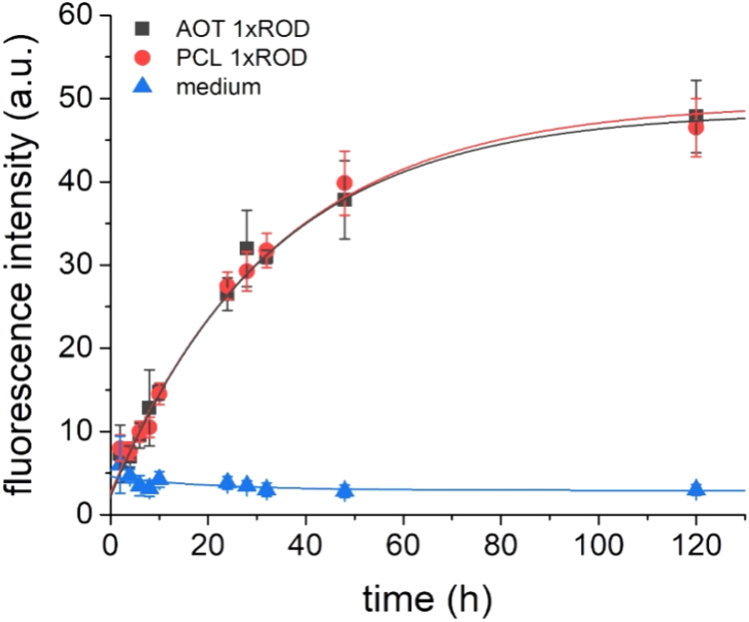
Profile of NC transfer through the artificial BBB model expressed as fluorescence intensity increase vs time of exposition for NCs.

We did not notice any meaningful differences in the penetration ability between the two studied types of NC cores. As expected, we noticed that fluorescence growth is the most rapid at the beginning of the experiment during the first 24 h, in which up to 60% of NCs cross the BBB model. After that time, the velocity of transfer slowly decreased, up to reaching a plateau at the end. Concerning the fact that both tested types of NCs show similar permeating profiles, it can be assumed that the transport mechanism across the BBB is the same. Our previous studies characterized the process of polymeric nanocapsule penetration through the hCMEC/D3 cell layer. Specifically, the expression of endothelial cell markers that characterize BBB cells (hCMEC/D3 cell line) was demonstrated. Moreover, using confocal microscopy, we visualized the ability of nanocapsules to penetrate the cell interior. By application of endocytosis inhibitors, it was shown that all studied nanocapsules undergo pinocytosis. Additionally, a time-dependent inhibition of the transcytosis process was observed after using filipin III, a caveolae-dependent transcytosis inhibitor. The results obtained in previous experiments indicated that PEGylated nanocapsules were the most efficient in the transcytosis experiment. Our results and other studies point to the hCMEC/D3 cell line as a suitable model for permeability studies.^13,53^ It has been reported that the size, shape, stiffness, and composition of nanoparticles impact their uptake and transport across the BBB, and in general, an inverse correlation between the nanoparticle size and its transport was suggested.^54^ Nanoparticles with the smallest size can easily penetrate the BBB and accumulate in the brain tissue.^55^ However, nanoparticles with sizes >500 nm can accumulate in the spleen and liver, while those smaller than <5 nm in diameter are excreted with urine.^56^ Thus, the nanoparticles with the size of 100–120 nm in diameter used in our study appear fairly optimal as potential carriers of CNS drugs. Very small and hydrophilic molecules are passing via the paracellular route via the tight junction complex, while at least partially lipophilic molecules could be taken the passive diffusion through the membrane phospholipid bilayer and cytoplasm. It should be underlined that PEGylated nanoparticles, as used in our study, is a strategy that has been used extensively to enhance nanoparticle interactions with biological materials. PEGylation can improve nanoparticle drug delivery by reducing charge interactions with the extracellular matrix and by preventing opsonization and phagocytosis by immune cells via either the microtubule-assisted endocytosis pathway or the paracellular pathway.^57–59^ It was reported that nanoparticles as large as 114 nm in diameter diffused within the human and rat brain, but only if they were densely coated with PEG. To this end, it was reported that 40 and 100 nm PEGylated nanoparticles spread rapidly within the brain tissue.^59^ Furthermore, PEG is considered a “stealth” molecule, which enables the nanoparticles to interact with endothelial receptors. It has been recognized that nanoparticle transport across biological barriers, like the endothelium including endothelial cells in tumors and the BBB, for nanoparticles above 200 nm in size is governed by micropinocytosis, whereas smaller nanoparticles (<200 nm in size) could be transported by micropinocytosis, as well as paracellular transport mechanisms. For example, it was found that PEGylated 130 nm nanoparticles are transported across lymphatic endothelial cells via micropinocytosis (likely clathrin-mediated endocytosis) and through paracellular transport routes.^60^ The detailed studies of this mechanism are outside the scope of this paper and should be an objective of further research.

This study showed that these newly designed polymeric theranostic NCs possess favorable physicochemical characteristics and an acceptable *in vitro* biosafety profile. The latter was evidenced by data showing that in a wide range of dilutions, the suspension of NCs did not deteriorate the viability of neuroblastoma SH-SY5Y or primary cortical neuronal cultures. These results are in agreement with previous reports that polymeric nanoparticles are biocompatible and may be quite useful for designing NCs for neuroprotective drugs.^61^ However, it should be mentioned here that the concentrated (10×) nanoparticle suspension, regardless of whether it contained Gd or not, significantly decreased the cell viability. The reason for the cytotoxic activity of the NC stock suspension is unknown, but it may be due to some residual chloroform from the emulsification process or free polymer chains left over from the LbL process. Nevertheless, upon dilutions, the toxic effect of the nanoparticle suspension on cell viability quickly vanished, and for further experiments, the nontoxic 40× dilution was mainly used. Since we aimed to design the NCs for putative neuroprotective agents that act intracellularly, namely, calcineurin inhibitors CsA and FK506, it was important to demonstrate that the NCs penetrate the cells. To this end, we showed an efficient and time-dependent uptake of rhodamine-labeled NCs of both types in SH-SY5Y cells. Our data indicate that the uptake of the NCs in the cells is not very rapid, as at 3 h of incubation, almost 40% of cells were still unlabeled with rhodamine. On the other hand, this experiment did not allow us to find out if the cell-uptaken NCs were able to release their cargo in order to exert a neuroprotective effect. Therefore, the fate of the designed nanotheranostic in the cells, their intracellular stability and distribution, and their neuroprotective effects against cell-damaging insults need to be evaluated in further studies. Since the calculated concentration of CsA and FK506 in the drug-loaded nanocore suspensions was 25 mg/L for both types of NCs, which roughly equals 21 μM CsA and 31 μM FK506, it may be suggested that even 100 times diluted NCs could still provide neuroprotective submicromolar concentrations of the calcineurin inhibitors. Indeed, Leventhal et al. (2000) found that *in vitro* CsA protected striatal neurons from 3-nitropropionic acid toxicity at lower (0.2 or 1.0 μM), but not at higher (5.0 μM) doses.^62^ Also, it was reported that CsA and its analogue in submicromolar concentrations prevented *in vitro* mitochondrial damage.^63^ The efficacy of low concentrations of CsA to obtain neuroprotective effects *in vitro* contrasts with the necessity of administering high doses of this drug in order to protect the brain *in vivo*. Thus, intracarotid infusion of 5–10 mg/kg CsA was required to protect hippocampal neurons in the model of 5 min gerbil brain ischemia,^64^ which most likely is due to poor penetration of CsA through the BBB.

The recent report strongly supports the view that polymeric nanoparticles containing CsA, which targets the mitochondrial permeability transition pore (mPTP), are viable means to prevent ischemia-induced brain damage.^65^ Our results obtained in the *in vitro* BBB model clearly indicate that both types of the designed NCs readily cross the barrier with almost identical kinetics.

All in all, the biological part of this study indicates that both solid core (PCL) and liquid (AOT) core polymeric-based theranostic NCs of neuroprotective drugs are equally safe to cells when properly diluted, readily penetrate in the cells, and cross the BBB with similar kinetics.

## 4 Conclusions

A method of preparing polymeric-based theranostic NCs of neuroprotective drugs was developed. The NCs were formed using a nanoemulsion template with further functionalization by the layer-by-layer approach. Proposed NCs can serve as an effective theranostic nanosystem that can be observed and monitored by MRI or optical imaging (fluorescence). Developed polymeric-based theranostic NCs of neuroprotective drugs are safe for cells, readily penetrate them, and cross the BBB. These beneficial properties of the designed NCs revealed in *in vitro* tests encourage us to evaluate their potential as nanotheranostics in *in vivo* models of brain ischemia, for example. Our current findings suggest that the developed gadolinium-labeled polyelectrolyte NCs hold significant promise as a platform for future theranostic applications.
